# Can home rehabilitation impact impulse oscillometry and lung ultrasound findings in patients with scleroderma-associated interstitial lung disease? A pilot study

**DOI:** 10.1186/s13104-022-06064-6

**Published:** 2022-05-15

**Authors:** Samantha Gomes de Alegria, Patrícia Frascari Litrento, Iasmim de Oliveira Farias, Thiago Thomaz Mafort, Agnaldo José Lopes

**Affiliations:** 1 Post-Graduation Programme in Medical Sciences, School of Medical Sciences, State University of Rio de Janeiro (UERJ), Boulevard 28 de Setembro, 77, Vila Isabel, Rio de Janeiro, 20551-030 Brazil; 2 Department of Pulmonology, Piquet Carneiro Policlinic, State University of Rio de Janeiro (UERJ), Avenida Mal. Rondon, 381, São Francisco Xavier, Rio de Janeiro, 20950-003 Brazil; 3 Post-Graduation Programme in Rehabilitation Sciences, Augusto Motta University Centre (UNISUAM), Rua Dona Isabel, 94, Bonsucesso, Rio de Janeiro, 21032-060 Brazil

**Keywords:** Scleroderma, Pulmonary function test, Small airway disease, Lung ultrasound

## Abstract

**Objective:**

Exercise has been demonstrated to be beneficial for improving physical capacity and quality of life in people with scleroderma, although knowledge of its impact on the respiratory system is limited. This study evaluated the impact of therapist-oriented home rehabilitation (TOHR) on impulse oscillometry (IOS) and lung ultrasound (LUS) findings in patients with scleroderma-associated interstitial lung disease (ILD).

**Results:**

Twelve women with scleroderma underwent spirometry, IOS, and LUS before and after performing TOHR. Regarding spirometry, a normal pattern and restrictive damage were observed in five (41.7%) and seven (58.3%) participants pre-TOHR and post-TOHR, respectively. For IOS, an abnormal result was detected in nine (75%) pre-TOHR participants and six (50%) post-TOHR participants. Heterogeneity of resistance between 4–20 Hz (R4-R20) > 20% of the predicted value was observed in eight (66.7%) pre-TOHR participants and three (25%) post-TOHR participants (*P* = 0.031). An abnormal LUS result was observed in nine (75%) participants both pre-TOHR and post-TOHR. The main change observed was B-lines > 2, which was noted in nine (75%) participants both pre-TOHR and post-TOHR. Our findings suggest that TOHR for women with scleroderma-associated ILD improves the resistance and reactance measured by IOS, including small airway disease.

*Trial Registration ClinicalTrials.gov ID: NCT05041868 Registered on: 13th September 2021.*

## Introduction

Scleroderma is a chronic, progressive, and autoimmune disease characterized by cutaneous fibrosis, vasculopathy, and visceral damage [1]. Because it is an incurable condition, people with scleroderma need strategies to help manage the disease, such as rehabilitation, which plays an important role in improving the results reported by patients [2]. Physical activity has attracted increasing interest in recent years as an adjuvant therapy for people with scleroderma. However, information on the efficacy of therapist-oriented home rehabilitation (TOHR) for the lungs is limited, which may be intensely compromised by excessive deposition of collagen fibres and fibroproliferative changes in the microvasculature [3]. In fact, lung parenchyma involvement manifesting as interstitial lung disease (ILD) is the main cause of death in scleroderma, followed by pulmonary vascular involvement due to pulmonary hypertension [4].

Small airway disease (SAD) is a common finding in scleroderma and is detected in up to two-thirds of cases [5]. Similarly, ILD is a frequent complication in scleroderma, with up to 80% of patients presenting with pulmonary fibrosis [6]. Although impulse oscillometry (IOS) and lung ultrasound (LUS) have been described as promising techniques to evaluate SAD and ILD, respectively, in patients with scleroderma [7], little is known about the effects of exercise on the measurements provided by these techniques. Exercise has been demonstrated to be safe and beneficial for improving physical capacity, vascular function, and quality of life in people with scleroderma [8, 9], although knowledge of its impact on the respiratory system is limited. The present study aimed to evaluate the impact of TOHR on IOS and LUS findings in patients with scleroderma-associated ILD.

## Main text

### Methods

This study used a quasiexperimental and pre-post design and evaluated 12 consecutive women aged > 18 years who were regularly seen at Pedro Ernesto University Hospital of the Rio de Janeiro State University (UERJ), Rio de Janeiro, Brazil, and met the diagnostic criteria for scleroderma [10]. All patients had scleroderma with associated ILD confirmed by computed tomography (CT) scan. Patients with acute or chronic respiratory disorders, patients with comorbidities unrelated to scleroderma, and patients who were unable perform functional tests were excluded. The protocol was approved by the local ethics committee, and all patients signed an informed consent form.

The participants were subjected to TOHR, which was performed three times per week for 12 weeks. After evaluation, each patient received a prescription for TOHR exercises in the form of a booklet describing all exercises and a video demonstrating how to improve accessibility. Each session included muscle strengthening (including respiratory muscles), aerobic resistance, and flexibility exercises lasting 60 min. The session began with 5 min of warm-up exercises, followed by 20 min of strengthening movements for large muscle groups and resistance exercises using light weights and functional movements. Subsequently, 10 min of postural control training was performed through proprioceptive exercises on the ground, followed by 20 min of aerobic training in functional circuits. Finally, 5 min of stretching and relaxation were performed using calisthenic exercises. The participants were contacted weekly by telephone by a physical therapist who monitored treatment progression. Home-level exercise intensity was monitored using Borg’s Perceived Exertion Scale < 4 and peripheral oxygen saturation > 92% as criteria; if these signs were not within the indicated range, the patient was instructed to discontinue TOHR and contact the physical therapist in order to restart TOHR after 30 min [11]. The participants were evaluated before and after 12 weeks of TOHR [12, 13].

IOS was performed using an impulse oscillometer (Quark i2m, Cosmed, Rome, Italy). During the IOS evaluation, the participants were instructed to remain seated, support the cheeks with their hands, wear a nose clip, and then breath normally for 40 s. The following resistive and reactive parameters were evaluated: respiratory system resistance (Rrs) at 4 Hz (R4), 6 Hz (R6), 10 Hz (R10), and 20 Hz (R20); the mean resistance between 4 and 20 Hz (Rm); the heterogeneity of resistance between 4–20 Hz (R4–R20); resonance frequency (Fres); and the area under the reactance curve (AX). The following values were considered abnormal: R4, R6, R10, and/or R20 ≥ 150% of the predicted value; Fres > 12 Hz; AX > 3.60 cm H_2_O/L/s; and R4-R20 > 20%, which was also used to diagnose SAD [14–16]. Immediately after a rest period of approximately 5 min from IOS performance, spirometry was performed on the Vitatrace VT 130 SL equipment (Codax Ltda, Rio de Janeiro, Brazil) using standardized recommendations [17] and national reference values [18].

Finally, the participants performed LUS in an Aplio XG device (Toshiba Medical Systems, Tokyo, Japan) coupled to a 7.5- to 10-MHz multifrequency linear transducer or a 3.5- to 5-MHz convex transducer in B mode. All LUS evaluations were performed by two examiners, and any disagreements were resolved by consensus. As no consensus has been established for the LUS protocol, signal capture was performed in six areas of each hemithorax as follows: two anterior, two lateral, and two posterior. For the evaluation of pathological LUS signs, we sought to identify B-lines (defined as hyperechoic narrow-base reverberation artefacts, which extend similar to a laser beam to the edge of the screen) > 2, coalescent B-lines, and subpleural consolidations [19]. Points were assigned to each of the six areas as follows to obtain the aeration score: B-lines > 2, 1 point; coalescent B-lines, 2 points; and consolidations, 3 points; the sum of all areas represented the aeration score [20].

Data analysis was performed using IBM SPSS Statistics version 26.0 software (IBM Corp., Armonk, NY, USA). The normality of the variables was evaluated by the Shapiro–Wilk test. Because of the nonparametric distribution of the data, pre- and post-TOHR measurements were compared by the Wilcoxon signed-rank test (numerical data) and the unilateral exact McNemar test (categorical data). The absolute delta was obtained by subtracting the post-TOHR value from the pre-TOHR value. The results are expressed as the median (interquartile ranges) or as numbers (frequencies). Statistical significance was considered *P* < 0.05.

### Results

Among the 12 women with scleroderma evaluated for inclusion in the study, three were excluded due to difficulty walking. The time since diagnosis was 9 (3–18.5) years, and none of the women had a history of smoking. The baseline characteristics of the scleroderma patients participating in the study are shown in Table 1.

**Table 1:** Characteristics of scleroderma patients participating in the study at baseline

Variables	
Demographic/anthropometric data	
Age (years)	51 (40–63)
BMI (kg/m^2^)	26.4 (24–31)
Clinical characteristics	
Limited cutaneous scleroderma (n, %)	7 (58.3)
Diffuse cutaneous scleroderma (n, %)	5 (41.7)
ILD (n, %)	12 (100%)
PH (n, %)	3 (25%)
Gastrointestinal symptoms (n, %)	6 (50%)
Renal crisis (n, %)	1 (8.3%)
Serology	
Anti-TOPO I positivity (n, %)	8 (66.7%)
Anti-RNAP III positivity (n, %)	3 (25%)
Anti-centromere positivity (n, %)	1 (8.3%)

Results expressed as the median (interquartile range) or number (%)

ILD: interstitial lung disease (diagnoses by computed tomography); PH: pulmonary hypertension; anti-TOPO I: antibodies against topoisomerase I; anti-RNAP III: antibodies against RNA polymerase III

**Fig. 1:**
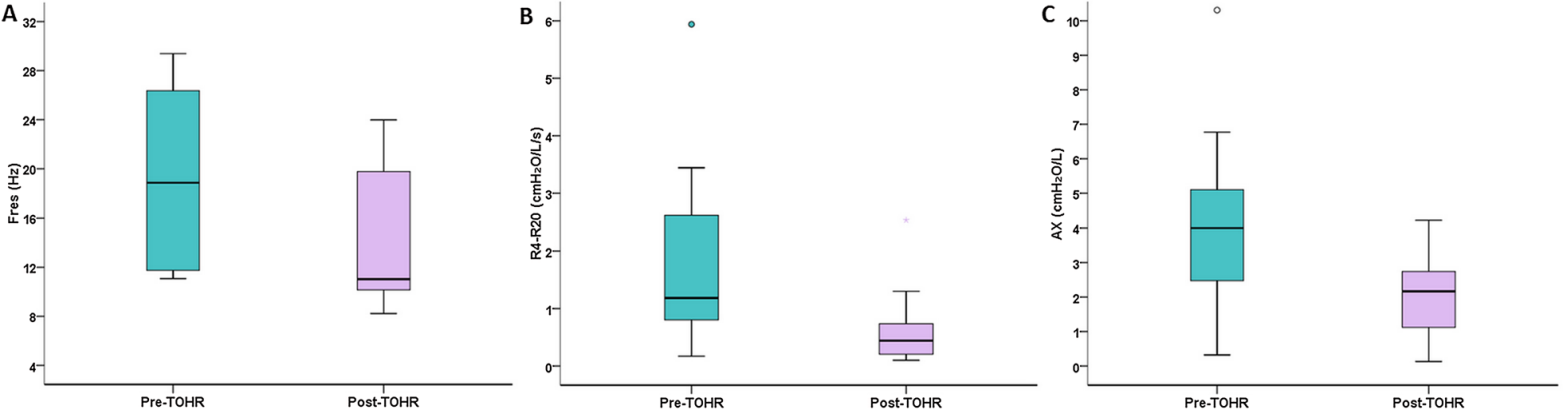
Changes in resonance frequency (Fres, *P* = 0.002) (**A**), the heterogeneity of resistance between 4–20 Hz, *P* = 0.021) (**B**), and the area under the reactance curve (AX, *P* = 0.002) (**C**) measured by impulse oscillometry between pre- and post-therapist-oriented home rehabilitation (TOHR)

Regarding pulmonary function tests (PFTs), a normal pattern and restrictive damage were observed in 5 (41.7%) and 7 (58.3%) participants, respectively, none of whom had obstructive damage on spirometry. This distribution of spirometric patterns was maintained after TOHR, although a discrete reduction in forced vital capacity between pre- and post-TOHR was noted (*P* = 0.06). Considering the changes in the resistive and reactive parameters on IOS, an abnormal test result was detected in 9 (75%) participants pre-TOHR and in 6 (50%) participants post-TOHR. Fres > 12 Hz was present in 8 (66.7%) and 5 (41.7%) participants, respectively, in the pre-TOHR and post-TOHR groups, while AX > 3.60 cm H_2_O/L/s was present in 7 (58.3%) and 2 (16.7%) participants, respectively, in the pre-TOHR and post-TORH groups. An R4-R20 value > 20% of the predicted value was observed in 8 (66.7%) participants pre-TOHR and in 3 (25%) participants post-TOHR (*P* = 0.031). Comparisons of the parameters provided by the PFTs pre- and post-TOHR are shown in Table 2 and Fig. 1.

**Table 2:** Pulmonary function parameters and lung ultrasound signals assessed pre- and post-therapist-oriented home rehabilitation

Variables	Pre-TOHR	Post-TOHR	Absolute delta	*P*-value
Spirometry				
FVC (% predicted)	72.6 (65–89)	74.5 (67–92)	1.9 (0.4–3.2)	0.06
FEV_1_ (% predicted)	71.3 (64.1–83.7)	74.1 (68–88)	2.8 (0.2–5.3)	0.07
FEV_1_/FVC (% predicted)	99.2 (95.8–102)	100 (99–104)	1.5 (0.1–3.5)	0.13
FEF_25-75%_ (% predicted)	67.3 (55.7–72.5)	77.1 (63–127)	6.5 (− 5.3 to 14.7)	0.24
Impulse oscillometry				
Fres (Hz)	18.9 (12–27)	11 (10–21)	− 5 (− 6.2 to − 1.3)	**0.002**
Rm (cmH_2_O/L/s)	4.44 (3.18–5.38)	3.94 (2.57–4.91)	− 0.58 (− 1.5 to 0)	0.091
R4 (cmH_2_O/L/s)	5.27 (4.07–7.97)	4 (2.85–6.03)	− 1.2 (− 4.3 to − 0.2)	**0.034**
R4 (% predicted)	150 (109–171)	126 (89–151)	− 18 (− 44.3 to − 4)	**0.021**
R6 (cmH_2_O/L/s)	4.66 (3.29–5.62)	3.82 (2.77–5.36)	− 0.86 (− 1.8 to − 0.1)	**0.034**
R6 (% predicted)	146 (109–170)	128 (87.8–149)	− 23.5 (− 33.8 to − 0.5)	**0.014**
R10 (cmH_2_O/L/s)	4.55 (3.24–5.41)	3.61 (2.69–4.92)	− 0.6 (− 2 to − 0.1)	**0.041**
R10 (% predicted)	144 (103–165)	126 (91.3–140)	− 14 (− 38.5 to − 3.5)	**0.025**
R20 (cmH_2_O/L/s)	4.45 (3.03–5.14)	3.55 (2.6–4.6)	− 0.6 (− 1.6 to 0.2)	0.099
R20 (% predicted)	128 (88–150)	117 (80–137)	− 13.5 (− 35 to − 12.3)	0.18
R4-R20 (cmH_2_O/L/s)	1.18 (0.79–2.72)	0.44 (0.2–0.79)	− 0.32 (− 2.2 to –0.03)	**0.021**
AX (cmH_2_O/L)	4 (2.33–5.22)	2.17 (0.79–2.80)	− 1.2 (− 3.1 to − 0.2)	**0.002**
Lung ultrasound				
B-lines > 2	3.5 (0.5–8.8)	2.5 (0.3–5.8)	− 1 (− 1.8 to 0)	0.07
Coalescent B-lines	0 (0–2.8)	0 (0–2.8)	0 (0–0)	1
Subpleural consolidations	0 (0–0)	0 (0–0)	0 (0–0)	1
Aeration score (points)	6 (0.5–15.8)	4.5 (0.25–13.5)	− 1 (− 1.8 to 0)	0.06

Results expressed as the median (interquartile range)

TOHR: therapist-oriented home rehabilitation; FVC: forced vital capacity; FEV_1_: forced expiratory volume in one second; FEF_25-75%_: forced expiratory flow during the middle half of the FVC; Fres: resonance frequency; Rm: mean resistance between 4–20 Hz; R4: resistance at 4 Hz; R6: resistance at 6 Hz; R10: resistance at 10 Hz; R20: resistance at 20 Hz; R4-R20: heterogeneity of resistance between 4–20 Hz; AX: area under the reactance curve

An altered LUS result was observed in 9 (75%) participants both pre-TOHR and post-TOHR. The main change observed was B-lines > 2, which was noted in 9 (75%) participants both pre- and post-TOHR. Coalescent B-lines and subpleural consolidations were present in 5 (41.7%) and 2 (16.7%) participants, respectively, both pre-TOHR and post-TOHR. However, a reduction in the aeration score between pre- and post-TOHR was identified, although without statistical significance (*P* = 0.06) (Table 2).

### Discussion

Rehabilitation is an important tool to help individuals manage scleroderma and potentially delay its disabling effects, including those involving the respiratory system [2]. One of the most important issues in the rehabilitation of patients with chronic diseases, including those with scleroderma, is regularity and repeatability, which can be more easily achieved by TOHR, especially during the current COVID-19 pandemic [3]. The main findings of the present study were improved resistive and reactive parameters measured by IOS after 12 weeks of TOHR, including SAD improvement. In this same period, no changes were observed in spirometric indices or LUS signals.

Although small airways are more susceptible to several pathogenic stimuli than larger airways, any obstruction must affect 75% of all small airways to be captured by traditional PFTs; therefore, the small airways have been labelled the “silent zone” of the lung [21]. In the present study, we used IOS—a noninvasive forced oscillation technique—to detect SAD because it has been advocated as a valuable tool for sensitive evaluations of SAD [7]. Interestingly, we observed that three-fourths of the patients had altered IOS, and more than two-thirds had SAD diagnosed by an increase in R4-R20. In line with our findings, Ostojic and Vujovic [5] detected SAD in 66.6% of patients with scleroderma, although they used the maximal expiratory flow at 25% of the forced vital capacity to diagnose SAD. Unlike these studies, Bonifazi et al. [7] used IOS and Silva et al. [22] used the nitrogen single-breath washout test and observed SAD in 21.5% and 28.8% of patients with scleroderma, respectively. These differences may be explained at least in part by the varied frequencies of interstitial involvement, as SAD may indicate possible prominent bronchiolar involvement in scleroderma-related ILD [5, 23].

Small airway involvement may have a considerable influence on the activities of daily living and on the perceptions of patients despite the limited involvement of the lung parenchyma detected by traditional PFTs [7]. In fact, one of the most striking findings of our study was the improvement in SAD post-TOHR. Yakut et al. [24] compared the effects of supervised exercise and a home exercise programme in patients with scleroderma for 12 weeks and observed that pulmonary function assessed by spirometry and by the diffusing capacity for carbon monoxide and the severity of dyspnoea improved significantly only in the group engaging in supervised exercises (*P* < 0.05). Notably, improvement in traditional PFTs has not been described in chronic lung diseases after rehabilitation [25]. Thus, we hypothesized that the improvement in SAD observed in our study may be related to changes at the bronchiolar level that are not captured by the PFTs routinely used in clinical practice.

We used LUS in the present study because in addition to safety, accessibility, execution speed, reproducibility, low cost, and good patient acceptance, it can be repeated in a very short period due to the absence of ionizing radiation and has a rate of concordance with CT greater than 80% for the detection of ILD [26]. B-lines, which are signs of increased density of the peripheral lung parenchyma with partial loss of aeration indicative of pulmonary interstitial syndrome, were present at baseline in approximately three-quarters of our patients [27, 28]. In our sample, coalescent B-lines were also frequent, which generally correspond to the ground-glass opacity pattern on CT and thus a high-grade interstitial syndrome [20]. Importantly, few changes in the aeration score between pre- and post-TOHR were found in our sample, indicating the irreversibility of LUS signals with the exercise programme implemented.

In conclusion, our preliminary findings suggest that TOHR improved the resistance and reactance (including SAD) measured by IOS in a sample of women with scleroderma-associated interstitial lung disease. However, TOHR did not cause any change in spirometric and ultrasound findings. These positive pulmonary function results are promising if we consider that they were obtained with a simple exercise programme that has the advantage of promoting physical activity by stimulating a healthier lifestyle and can serve as a useful support for pharmacological therapies.

## Limitations

Our study also has limitations. First, the study involved a small number of women. Second, we only evaluated women in our sample. However, most scleroderma patients are women, with a ratio of approximately 8:1 [29], and sex accounts for 30% of the variation in lung function, thus supporting sex-differentiated interpretive strategies [30, 31]. Third, we did not assess exercise tolerance and quality of life, although exercise has previously been demonstrated to be beneficial for these measures in people with scleroderma [32, 33]. Fourth, LUS has poor specificity and does not provide information on pulmonary morphology, and these findings need to be confirmed by CT. Whether the LUS is sufficiently sensitive to detect changes in the extent of lung disease over time remains to be determined in large samples. Because of advances in lung elastography (LEG) in recent decades, interest in its use to provide a qualitative and quantitative assessment of tissue elasticity has been increasing. Combining LUS with LEG could provide a more robust, quantitative method for evaluating superficial lung stiffness [34, 35]. A prospective trial with a larger number of individuals with scleroderma who can be compared with a control group is necessary to generalize our results.

## Data Availability

The datasets used and/or analysed during the current study available from the corresponding author on reasonable request.

## Abbreviations

AX: Area under the reactance curve
CT: Computed tomography
Fres: Resonance frequency
ILD: Interstitial lung disease
IOS: Impulse oscillometry
LEG: Lung elastography
LUS: Lung ultrasound
PFT: Pulmonary function test
Rm: Mean resistance between 4–20 Hz
Rrs: Respiratory system resistance
R4–R20: Heterogeneity of resistance between 4–20 Hz
SAD: Small airway disease
TOHR: Therapist-oriented home rehabilitation
